# Combination of Morroniside and Diosgenin Prevents High Glucose-Induced Cardiomyocytes Apoptosis

**DOI:** 10.3390/molecules22010163

**Published:** 2017-01-19

**Authors:** Wen-Xia Pi, Xiao-Peng Feng, Li-Hong Ye, Bao-Chang Cai

**Affiliations:** State Key Laboratory Cultivation Base for TCM Quality and Efficacy, College of Pharmacy, Nanjing University of Chinese Medicine, Nanjing 210023, Jiangsu, China; fxpen@sina.com (X.-P.F.); cocolihongye@126.com (L.-H.Y.); bccai@126.com (B.-C.C.)

**Keywords:** morroniside, diosgenin, cardiomyocytes, diabetic cardiomyopathy, Bcl-2/Bax/caspase-3 signaling pathway

## Abstract

*Cornus officinalis* and *Dioscorea opposita* are two traditional Chinese medicines widely used in China for treating diabetes mellitus and its complications, such as diabetic cardiomyopathy. Morroniside (Mor) of *Cornus officinalis* and diosgenin (Dio) of *Dioscorea opposita* formed an innovative formula named M + D. The aims of the present study were to investigate myocardial protective effect of M + D on diabetic cardiomyopathy (DCM) through the inhibition of expression levels of caspase-3 protein, and identify the advantage of M + D compared with Mor, Dio, and the positive drug metformin (Met). We detected cell viability, cell apoptosis, intracellular reactive oxygen species (ROS) levels, and the expression levels of Bcl-2, Bax, and caspase-3 protein in rat cardiomyocytes. In result, Mor, Dio, and M + D increased cell viability, inhibited cell apoptosis and decreased ROS levels. Additionally, the expression of Bax and Bcl-2 protein was modulated and the expression levels of caspase-3 protein were markedly decreased. Among the treatment groups, M + D produced the most prominent effects. In conclusion, our data showed for the first time that Mor, Dio, and M + D prevented high glucose (HG)-induced myocardial injury by reducing oxidative stress and apoptosis in rat cardiomyocytes. Among all the groups, M + D produced the strongest effect, while Mor and Dio produced weaker effects.

## 1. Introduction

Diabetic cardiomyopathy (DCM), also known as diabetic heart disease, was originally put forward by Rubler in 1972 [1]. In recent years, DCM has imposed an increasing social and economic burden with an increase in the number of diabetics. Increasing evidence demonstrates that hyperglycemia induces apoptotic of cardiomyocytes, resulting in the development of DCM through different mechanisms [2,3], including oxidative stress and the Bax pathway. On the one hand, hyperglycemia may induce excessive production of reactive oxygen species (ROS) that is regarded as the most important contributor in the occurrence and progression of DCM [4], causing oxidative stress and eventually promoting cell apoptosis. Recent studies suggested that accumulation of advanced glycation end-products (AGEs) under hyperglycemic conditions contributed to the development of diabetic complications. AGEs exert their effects directly or by binding their member receptor, receptor for advanced glycation end-products (RAGE), to activate the downstream signaling pathway [5]. Mitogen activated protein kinase (MAPK) is an essential intracellular signaling pathway involved in cell growth, apoptosis, and a series of physiological and pathological processes [6]. Advanced glycation end products-Receptor for advanced glycation end products (AGE-RAGE) interaction can induce pathophysiological cascades linked to the downstream activation of NF-κB, which in turn leads to ROS generation [7], and enhance the phosphorylation of p38 MAPK, which can lead to the apoptosis of cardiomyocytes [8]. On the other hand, hyperglycemia also can lead to cell apoptosis through activation of Bax signaling pathway.

Traditional Chinese medicine (TCM), has been attracting more and more attention because of its prominent advantages, stable curative effects, and low toxicity in comparison to Western medicines [9,10]. *Cornus officinalis Sieb.et Zucc*, (Cornaceae), a deciduous tree native to the Eastern Asia, distributed mainly in China, as well as Korea and Japan, known as “Shanzhuyu” in Chinese, often appeared in traditional Chinese medicine formulations, such as liu-wei-di-huang pills, for the treatment of diabetes [11]. It has also been reported that morroniside was a major active component of *Cornus officinalis* and had a markedly anti-diabetic effect via reducing oxidative stress, inflammation, and apoptosis [12]. Moreover, previous study from our lab has demonstrated that morroniside had a protective effect on high glucose-induced cardiomyocyte apoptosis [13]. The content of morroniside from the iridoid glycoside fraction of *Cornus officinalis* (219.5 mg/g) is the highest. So morroniside is regarded as main active components in *Cornus officinalis* [14]. Wang et al. research showed that the activity of SOD and the content of glutathione were significantly improved. Additionally, expression of caspase-3 in rat ischemic cortex tissue was decreased after treatment with morroniside [15]. *Dioscorea opposita* Thunb, (Dioscoreaceae), known as “Shanyao” in Chinese, is classified as medicinal and edible plant in traditional Chinese medicine [16] and also used as herb pairs of *Cornus officinalis* in the prescriptions of traditional Chinese medicine dictionary for the treatment of diabetes [17]. Literature has reported that diosgenin was major a steroidal saponin in yam and the main metabolite of the steroidal saponins in vivo [18]. It had significantly protective effect on diabetes by attenuating the activities of carbohydrate metabolic enzymes [19]. In addition, diosgenin has potential effects on cardiovascular diseases and insulin secretion in STZ induced diabetic rats [20]. However, the protective effect of combination of morroniside and diosgenin on DCM has so far not been experimentally researched. Therefore, it aroused our interests to explore if the protective effect of combination of Mor and Dio played a crucial role on cardiomyocytes via the underlying mechanisms of multi-components on multi-targets during the development and progression of DCM.

We hypothesized that M + D exerted their myocardial protective effect on DCM through activation of the Bax signaling pathway. Furthermore, the advantage of M + D compared with Mor, Dio and the positive drugs (Met) would be discussed. To prove this hypothesis, we detected cell viability and apoptosis by MTT assay and TUNEL staining, ROS by using the fluorescent probe dichlorofluorescein diacetate bis (DCFH-DA), Bcl-2/Bax, and the expression levels of caspase-3 protein by Western blotting.

## 2. Results

### 2.1. Effects of (M + D)H on Cell Viability

**Figure 1:**
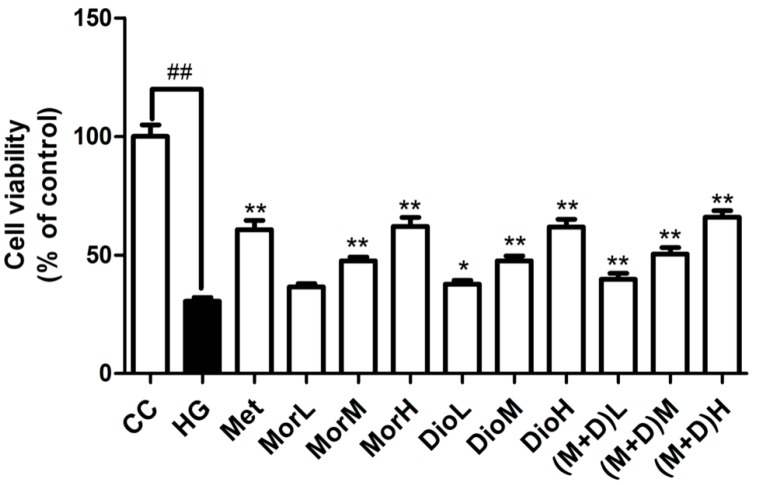
Effects of Mor (25, 50, 100 μg/mL), Dio (25, 50, 100 μg/mL), and M + D (25, 50, 100 μg/mL) on rat cardiomyocyte viability under HG-induced cell injury after pretreated 72 h. Values are expressed as the mean ± SD. ^##^ *p* < 0.01 vs. CC; ** *p* < 0.01, * *p* < 0.05 vs. HG.

Cell viability is shown in Figure 1 using the MTT assay. At 72 h after high glucose injury in cardiomyocytes, in HG group, the proliferation of cardiomyocytes was markedly decreased compared with the CC group. However, in the MorH group (100 μg/mL), the DioH group (100 μg/mL), and the (M + D)H group (100 μg/mL), the proliferation of cardiomyocytes was significantly increased compared to that in the HG group (*p* < 0.01). There was no obvious statistical difference between (M + D) group and Mor or Dio groups. The results of the MTT assay showed that MorH, DioH, and (M + D)H had an obviously protective effect on high glucose-induced cardiomyocytes injury. Then, MorH, DioH, and (M + D)H were, therefore, mainly used in subsequent experimental comparisons.

### 2.2. Effects of (M + D)H on Cell Apoptosis

**Figure 2:**
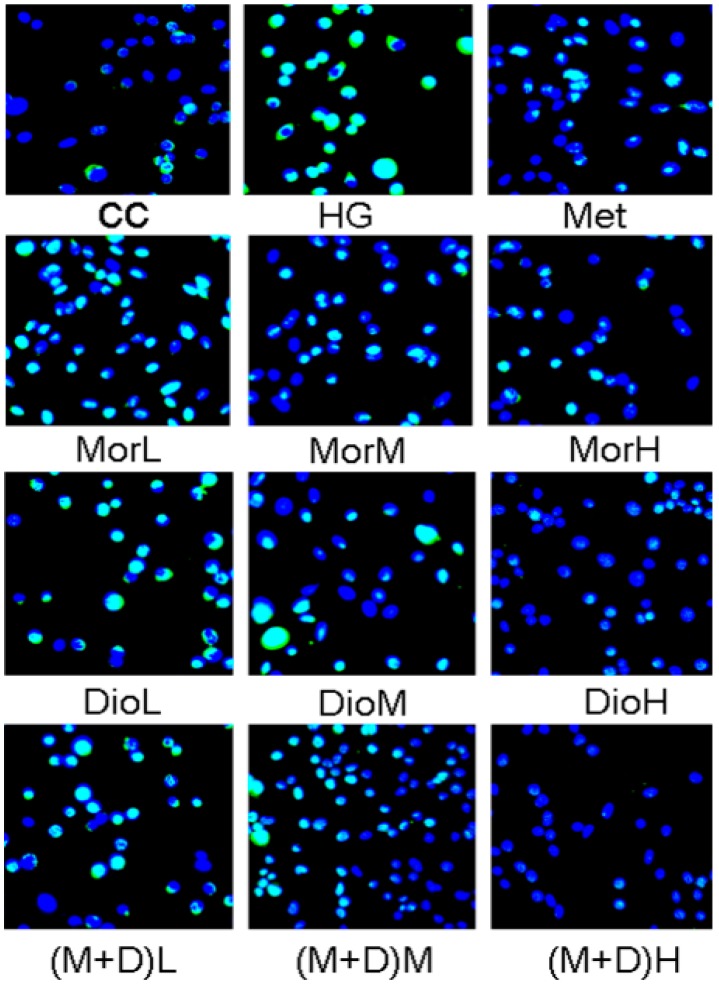

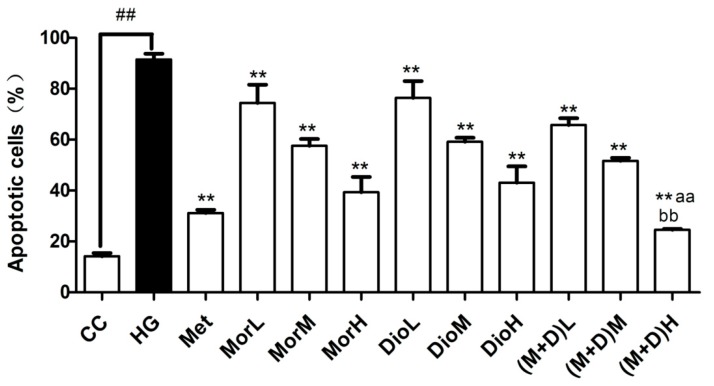
Effect of Mor (25, 50, 100 μg/mL), Dio (25, 50, 100 μg/mL), and M + D (25, 50, 100 μg/mL) on HG-induced cardiomyocyte apoptosis. (M + D)H significantly prevents from cardiomyocytes apoptosis as determined with TUNEL staining. Data are expressed as mean ± SD. ^##^ *p* < 0.01 vs. CC; ** *p* < 0.01, vs. HG; ^aa^ *p* < 0.01, vs. MorH; ^bb^ *p* < 0.01, vs. DioH.

Apoptosis assay was measured with TUNEL staining. Cells with green nuclei were considered apoptotic, and our results revealed that few cells with nuclei stained green were observed in the (M + D)H group (Figure 2). After being exposed to HG for 48 h, compared with the CC group, the total cell numbers were significantly reduced, and approximately 91.53% of cells showed apoptotic hallmarks. Compared with the HG group, (M + D)H significantly reduced the percentage of apoptotic cells to 24.59% (*p* < 0.01, Figure 2), so that the total cell numbers were significantly increased. It was found that Mor, Dio, and M + D could reduce apoptosis of cells to varying degrees. Based on the comparison, (M + D)H was more effective than MorH and DioH in inhibiting apoptosis of cardiomyocytes under hyperglycemic conditions.

### 2.3. Effects of (M + D)H on Intracellular Reactive Oxygen Species (ROS)

**Figure 3:**
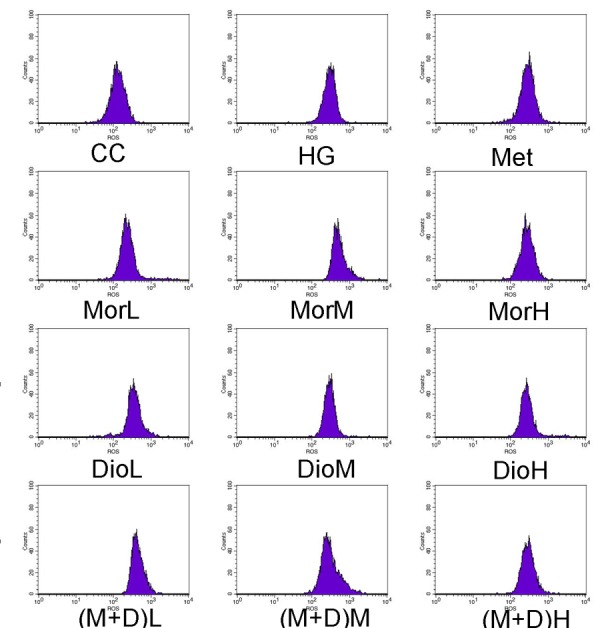

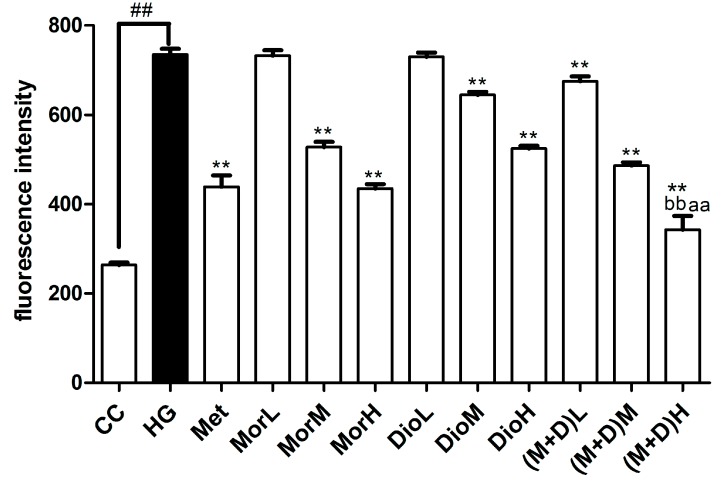
(M + D)H protects cardiomyocytes from mitochondrial production ROS induced with HG as determined with DCFH staining. Data are expressed as mean ± SD. ^##^ *p* < 0.01 vs. CC; ** *p* < 0.01, vs. HG; ^aa^ *p* < 0.01, vs. MorH; ^bb^ *p* < 0.01, vs. DioH.

The molecular probe DCFH-DA was used to label peroxide in cardiomyocytes. After DCFH-DA, the results revealed that the ROS levels of the HG group were increased to 735.00 ± 12.82% as compared to the control level (263.75 ± 5.24%, *p* < 0.01), yet pretreatment of the cells with (M + D)H (342.51 ± 31.40%) markedly reduced the ROS levels compared with MorH (434.50 ± 10.14%), DioH (524.57 ± 5.73%), and Met (*p* < 0.01 Figure 3). There was no significant difference between Mor or Dio and HG group. Therefore, it was concluded that the cardioprotective effect of M + D was significant and enhanced its antioxidant effect.

### 2.4. Effects of (M + D)H on the Expression Levels of Bax and Bcl-2 Protein

**Figure 4:**
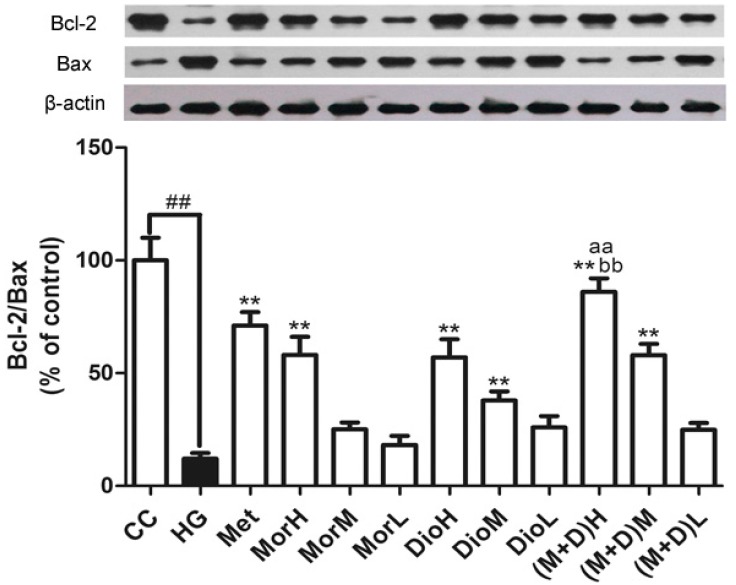
Regulation of proteins Bcl-2 and Bax associated with apoptosis by MorH, DioH, and (M + D)H. Data are expressed as mean ± SD. ^##^ *p* < 0.01 vs. CC; ** *p* < 0.01, vs. HG; ^aa^ *p* < 0.01, vs. MorH; ^bb^ *p* < 0.01, vs. DioH.

To determine whether apoptosis of cardiomyocytes was involved in the Bax pathway, we tested expression levels of Bax and Bcl-2 protein. As shown in Figure 4, the Bcl-2/Bax ratio was decreased significantly in the HG-treated group compared with untreated controls. In contrast, the Mor, Dio, and Met treatment increased the ratio of Bcl-2 to Bax in cardiomyocytes to different degrees. In the (M + D)H-treated group, the ratio of Bcl-2 to Bax was markedly increased compared to MorH, DioH, and Met (*p* < 0.01). Altogether, these data suggested that (M + D)H was the most effective in the aspect of anti-apoptosis via the Bax pathway.

### 2.5. Effects of (M + D)H on the Expression Levels of Caspase-3 Protein

**Figure 5:**
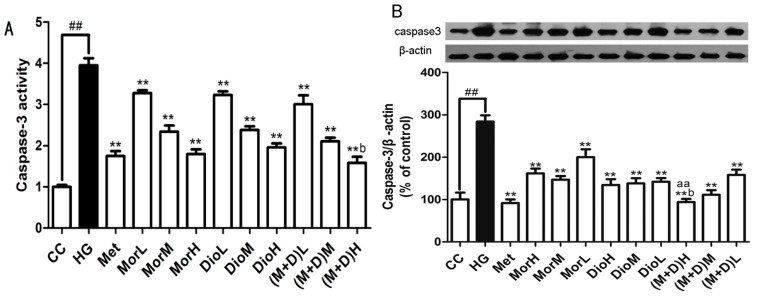
(**A**) Effect of (M + D)H on caspase-3 activity by using a colorimetric activity assay kit. Data are expressed as mean ± SD. ^##^ *p* < 0.01 vs. CC; ** *p* < 0.01 vs. HG; ^aa^ *p* < 0.01, vs. MorH; ^b^ *p* < 0.05 vs. DioH; and (**B**) the effect of (M + D)H on the expression levels of caspase-3 protein of cardiomyocytes after HG injury. Data are expressed as mean ± SD. ^##^ *p* < 0.01 vs. CC; ** *p* < 0.01 vs. HG; ^aa^ *p* < 0.01, vs. MorH; ^b^ *p* < 0.05 vs. DioH.

It is known that HG-induced apoptosis of cardiomyocytes is tightly regulated by caspase-3 [21]. Hence, we measured the expression levels of caspase-3 protein in cardiomyocytes by Western blot. As shown in Figure 5B, HG treatment significantly increased the expression levels of caspase-3 protein. In contrast, cardiomyocytes that were pre-treated with Mor, Dio, M + D and Met exhibited a significant decrease in expression levels of caspase-3 protein compared with the HG-treated cells at the same time point. However, (M + D)H was the most effective compared to MorH, DioH, and Met (*p* < 0.01). These results indicated that the cardioprotective effect of M + D better inhibited the expression levels of caspase-3 protein through blocking a Bax pathway.

### 2.6. Effects of (M + D)H on Caspase-3 Activity

To further confirm whether M + D could inhibit caspase-3 activity, we examined caspase-3 activity by using a colorimetric activity assay kit. As illustrated in Figure 5A, HG resulted in a prominent increase in caspase-3 activity compared to that in the CC group (*p* < 0.01), whereas the Mor, Dio, and Met reduced the level of caspase-3 activity to varying degrees compared with that of the HG group (*p* < 0.05). However, in the (M + D)H group, caspase-3 activity can be significantly inhibited compared to that in MorH and DioH group. These data indicated that apoptosis of cardiomyocytes was involved with caspase-3 activity regulated by the Bax pathway.

### 2.7. Discussion

Diabetic cardiomyopathy is characterized by hypertrophy and apoptosis of cardiomyocytes. Numerous mechanisms have been considered to be implicated in the pathogenesis of DCM, such as oxidative stress [22]. It has been confirmed that hyperglycemia can generate ROS through the formation of advanced glycation end products (AGEs) [23], which may eventually lead to cardiomyocyte death [21]. Moreover, considerable evidence suggests that overproduction of ROS induced by hyperglycemia is a crucial factor in the development of diabetic cardiomyopathy (DCM) [24,25]. A series of biological damage caused by ROS is a primary contributor to chronic diseases, such as DCM. Accordingly, we observed that the change of ROS among treatment with HG and treatment with Mor, Dio, and M + D. The results indicated the ROS levels in (M + D)H group were obviously decreased, which suggested that M + D could prevent the apoptosis of cardiomyocytes through the effect of the antioxidant, meanwhile, further validating that the combination of Mor and Dio produced greater effects than each component alone.

There is numerous evidence to manifest that apoptosis play an important role in the development of diabetic cardiomyopathy (DCM) [26]. Cardiomyocyte apoptosis is one of the major contributors to the development of myocardial infarcts [27,28]. In the present study, cardiomyocytes survival and apoptosis rate were monitored through MTT assay and TUNEL staining. These results strongly suggested that M + D had obvious anti-apoptotic effects. However, myocardial apoptosis is a complicated process that is mediated by a series of enzymes and numerous molecules, including the release of cytochrome c, the opening of the mitochondrial permeability transition pore, and Bax pathways that can regulate the expression levels of caspase-3 protein [29]. Caspase-3 is considered to be activated during the final step of the proapoptotic signaling pathway in many cell lines [30]. In addition, caspase-3 activity can be induced by the proapoptotic Bax family proteins and inhibited by the anti-apoptotic Bcl-2 family proteins. Bax can neutralize Bcl-2 actions by forming heterodimers with Bcl-2 [31,32]. Of course, previous investigations have also shown that a decrease in anti-apoptotic Bcl-2 family proteins and an increase in proapoptotic Bax family proteins are associated with the process of apoptosis [33]. Thus, we monitored the expression levels of caspase-3, Bcl-2, and Bax protein. In the current study, we found that M + D significantly inhibited the cell apoptosis through suppressing the expression of Bax proteins, and increasing the expression of Bcl-2 proteins so as to maintain the ratio of Bcl-2 to Bax balance, which further demonstrated that the combination of Mor and Dio produced the greatest protective effect on DCM via the Bax signaling pathway. Additionally, there are also studies demonstrating that overproduction of ROS increases the Bax/Bcl-2 expression ratio, which can decrease mtPTP and subsequently mediate the expression levels of caspase-3 [34,35]. Therefore, we also concluded that M + D exerted its anti-apoptotic effect by eliminating ROS production.

It is reasonable to speculate that this result could be mainly attributed by different molecular mechanisms of Mor and Dio. Meanwhile, these findings may also provide valuable insight on the possible effects and use of M + D as a feasible therapeutic option for the treatment of diabetic cardiomyopathy. In addition, we will do a direct comparison of the activity of the plant extract and the pure compound and further confirm the effect of M + D. Furthermore, we will do further study to perform a quantitative analysis of the drug combination and compare different concentrations and proportions of Mor + Dio.

## 3. Materials and Methods

### 3.1. Chemicals and Reagents

Metformin hydrochloride tablets were obtained from Shenzhen Zhonglian Pharmaceutical Co., Ltd. (Shenzhen, China). Morroniside (molecular formula: C_27_H_42_O_3_, molecular weight: 414.62, purity > 98%) and Diosgenin (molecular formula: C_17_H_26_O_11_, molecular weight: 402.38, purity > 98%) were purchased from a Shanghai source, Biological Technology Co. Ltd. (Shanghai, China). Their quality control data was provided by normalization of the peak areas detected by high performance liquid phase (HPLC). Trypsin-Ethylenediaminetetraacetic acid (EDTA) digestive juice (KGY001), green-streptomycin mixture, phosphate-buffered saline (PBS), Total protein extraction kit (KGP250), sodium dodecyl sulfate polyacrylamide gel electropheresis (SDS-PAGE) gel electrophoresis kit, Western blotting test kit (KGP1201), ECL test kit (KGP1123), Bradford protein assay kit, pre-stained protein molecular weight (KGP441) and Ponceau S solution were obtained from Nanjing Kaiji Biological Technology Development Co. Ltd. (Nanjing, China). Six-well cell culture plates, 24-well cell culture plates, 96-well cell culture plates, fetal bovine serum (FBS), RPMI-1640, and a Transwell chamber were purchased from Corning Incorporated (New York, NY, USA). Metrigel and Crystal Violet (C3886) were purchased from Sigma Co. (St. Louis, MO, USA). Dimethyl Sulphoxide (DMSO) was provided by Shanghai Long billion Chemical Reagent Co., Ltd. (Shanghai, China). Color developing solution and Fixer were obtained from Wuxi City Qi Ling Service Supplies Factory (Wuxi, China). All antibodies were obtained from Nanjing Kaiji Biological Technology Development Co. Ltd. (Nanjing, China).

### 3.2. Primary Culture of Rat Cardiomyocytes

In this experiment, neonatal rat cardiomyocytes were prepared from 1–3 day old Sprague-Dawley rats as previously described [36]. The hearts of neonatal SD rats were dissected and digested with 0.125% trypsin until a single cardiomyocyte was obtained. Then the dissociated cells were maintained in Dulbecco’s modified Eagle’s medium (DMEM) supplemented with 10% FBS at 37 °C, and incubated in 5% CO_2_ incubator.

### 3.3. Grouping

After 24 h cultured, rat cardiomyocytes were randomly divided into twelve groups: (1) control group without any treatment (CC); (2) high glucose model group (HG), pretreated with glucose (30 mM); (3) the positive group (Met), pretreated with metformin; (4–6) Morronisid group (Mor), pretreated with Mor at concentrations of 25, 50 or 100 μg/mL, which were respectively called MorL, MorM or MorH; (7–9) Diosgenin group (Dio), pretreated with Dio at concentrations of 25, 50, or 100 μg/mL, which were called DioL, DioM, or DioH; (10–12) M+D group, pretreated with Mor (25 μg/mL), then cardiomyocytes were pretreated with Dio (25 μg/mL), which were called (M + D)L (25 μg/mL), (M + D)M (50 μg/mL), or (M + D)H (100 μg/mL). The cells were pretreated with Mor, Dio, (M + D), and Met for 12 h before incubation with 30 mM glucose for 72 h. The cardiomyocytes incubation to morronisid and diosgenin is concomitant.

### 3.4. Analysis of Cell Viability by MTT Assay

Cardiomyocytes were seeded on 96-well plates at a density of 5 × 10^4^ cells/mL in 100 μL DMEM per well and incubated at 37 °C in 5% CO_2_ incubator for 24 h. Then the culture medium was discarded, and the cells were washed twice with phosphate-buffered saline (PBS). After different treatment, the 96-well plates were incubated at 37 °C in 5% CO_2_ incubator for 72 h. Finally, 20 μL of the MTT solution (5 mg/mL) was added into each well and the plates were incubated for 4 h. After that, the medium was removed and DMSO (150 μL) was added into each well. The optical density (OD) value was determined spectrophotometrically at 490 nm with a microplate reader.

### 3.5. TUNEL Staining

Cardiomyocytes were evaluated for apoptosis by the terminal deoxynucleotidyl (TUNEL) method, using a commercially available kit according to the manufacturer’s instructions. The rat cardiomyocytes were then washed twice with phosphate-buffered saline (PBS) and observed by a fluorescence microscopy (Leica, Wetzlar, Germany).

### 3.6. Detection of Intracellular Reactive Oxygen Species

After the addition of DCFH-DA to a final concentration of 10 μM, rat cardiomyocytes divided into groups were placed in a 37 °C at 5% CO_2_ incubator for 30 min. Then the cells were washed twice with PBS and digested with 0.25% trypsin. After washed twice with PBS again, the cells were plated in 96-well plates with 500 μL cell suspension. The fluorescence intensity (excitation wavelength 488 nm and emission wavelength 525 nm) was detected and analyzed by fluorescence enzyme labelling.

### 3.7. Western Blot Analysis

The cells were lysed with cold lysis buffer and the proteins were electrophoresed on 10% SDS-PAGE gel and transferred onto polyvinylidene difluoride membrane [37]. The membranes were blocked in 5% non-fat milk and then were incubated with the following antibodies: Bcl antibodies (1:5000), Bax antibodies (1:5000), and caspase antibodies (1:5000). Then, the membranes were incubated in 5% milk overnight at 4 °C. After washing the membranes three times for 10 min in TBS-T, Primary antibody was removed and incubated with a horseradish peroxidase-conjugated secondary antibody IgG (BA1054, 1:5000 dilution) for 1–2 h. After washing the membranes three times in TBS-T again, the antigen–antibody bands were detected with enhanced chemiluminescence reagent kit and Images were taken using the ChemiDoc XRS system with Quantity One software (Bio-Rad, Richmond, CA, USA).

### 3.8. Caspase-3 Activity Assay

Caspase-3 activity was measured using a colorimetric activity assay kit (Nanjing biological science and Technology Development Co., Ltd., Nanjing, Jiangsu, China) according to the manufacturer’s instructions. In brief, rat cardiomyocytes in experimental groups were lysed in ice-cold lysis buffer, placed on ice for 30 min, and then centrifuged at 4 °C for 15 min at 16,000 rpm. After determining the protein concentration, the supernatant was incubated with the caspase-3 substrate (Ac-DEVD-PNA) on a 96-well-plate. The activity of caspase-3 was detected at 405 nm by using a microplate reader (Biotek Synergy HT, Winooski, VT, USA).

### 3.9. Statistical Analysis

Data were presented as mean ± standard deviation (SD). Statistical analysis was performed using standard statistical methods (Graph-Pad Prism 5.0, GraphPad Software, San Diego, CA, USA). Significant differences (*p* < 0.05) among groups were determined by unpaired Student’s *t* test. One-way ANOVA followed by the Student–Newman–Keuls test was used to compare the multiple treatment conditions.

## 4. Conclusions

In conclusion, we found that M + D exerted a profound cardioprotective effect against HG-induced myocardial injury. The underlying mechanisms of M + D-mediated cardioprotection appear to be largely dependent on the regulating of expression of Bcl-2 and Bax protein. Our study not only provides insights into the cardioprotective effect of M + D, but also profoundly clarifies the widely applicable development strategy of traditional Chinese medicines. However, details of the molecular pathways and the therapeutic method of “multi-target, multi-drug” need further study. In addition, the cardioprotective effect of M+D would be further investigated by pharmacodynamics and pharmacokinetics in vivo. Meanwhile, it will be investigated that whether the protective effect of *Cornus officinalis* and *Dioscorea opposita* on DCM is only related to combination of Mor and Dio via metabonomics in vivo. Furthermore, we will conduct further study to confirm the possible synergistic effects between Mor and Dio by calculating the combination index according to standard equations.

## Abbreviations

DCM	diabetic cardiomyopathy
ROS	reactive oxygen species
SOD	superoxide dismutase
